# Abdominal Etching with Renuvion Helium Plasma

**DOI:** 10.1093/asjof/ojae007.074

**Published:** 2024-04-12

**Authors:** Dieter Brummund, Tarik Husain

**Affiliations:** Larkin Community Hospital, Miami, FL; Larkin Community Hospital, Miami, FL; Dr. Miami Beach, Miami Beach, FL

## Abstract

**Goals/Purpose:**

Differential liposuction with abdominal etching is a powerful technique in high definition liposculpture to achieve an athletic aesthetic. Etching is performed in a superficial plane to create indentures mirroring the dermal-fascial inscriptions of the underlying desired musculature. Renuvion radiofrequency-helium plasma energy (Apyx Medical, Clearwater, FL) is an emerging technology that is used for subdermal coagulation and contraction of the subcutaneous tissue. This case series describes combination of differential liposuction with abdominal etching and Renuvion helium plasma subdermal coagulation in 68 patients and evaluates the outcomes.

**Methods/Technique:**

A retrospective chart review of 68 patients undergoing differential liposuction with abdominal etching and Renuvion helium plasma subdermal coagulation between 2021 to 2023 by the senior author. The preoperative evaluation, markings, surgical technique and postoperative care are discussed. Clinical parameters assessed include age, gender, body mass index, combination of procedures, volume of lipoaspirate, degree of etching (light, moderate, deep), postoperative changes in weight, and complications such as contour irregularity, wound dehiscence, seroma, surgical site infection, patient dissatisfaction and operative revision.

**Results/Complications:**

68 consecutive patients who underwent abdominal etching with Renuvion by the senior author between July 2021 and August 2023 were included in this case series. There were 29 males (43%) and 39 females (57%). The average age of the patients was 39.11 (range, 22 to 64 years), with a non obese, average body mass index of 27.65 kg/m2 (range, 20.8 to 33.9 kg/m2). The average estimated intraoperative blood loss of patients was 104.68 ml (range, 75 to 150 ml); with an average tumescence of 4600 ml (range, 1400 to 8000 ml). The average lipoaspirate was 3920 ml (range, 500 to 9000 ml). In addition to the abdominal region, other areas liposuctioned at the time of etching included flanks (68, 100%), back (66, 97.05%), thighs (14, 20.58%), arms (9, 13.23%), neck (9, 13.23%), chest (8, 11.76%), axilla (3, 4.41%), calves (1, 1.47%). Use of ultrasound was used in 6 cases (8.82%), which were revisionary liposuction. Of our 68 abdominal etching with Renuvion cases the most common combined procedure was autologous fat transfer (20, 29.41%), platysmaplasty (4, 5.88%), mastopexy (5, 8%), breast augmentation (4, 5.88%), buccal fat pad excision (3, 4.41%), umbilical hernia repair (1, 1.47%) and rhytidectomy (1, 1.47%). There were 5 cases of seroma (7.35%) and 4 cases of contour irregularity (5.88%). There were 3 cases of surgical site infection (4.41%), but all in the area of fat grafting and not in the area of liposculpture. No cases of hyperpigmentation were identified. No major complications occurred including skin burns, skin necrosis, visceral injury, deep vein thrombosis, or pulmonary embolism. Overall 66 patients (97.05%) were satisfied following their procedure. Operative revision occurred in two patients (2.94%), who had residual skin laxity following Renuvion and desired skin excision.

**Conclusion:**

Differential liposuction with abdominal etching and Renuvion helium plasma are safe and effective in combination to synergistically achieve an athletic physique and enhance abdominal aesthetics. Complication rates are low with appropriate surgical technique. Almost all patients reviewed were satisfied with the procedure and maintained long-term results.

